# miR-155 Regulates the Proliferation of Glioma Cells Through PI3K/AKT Signaling

**DOI:** 10.3389/fneur.2020.00297

**Published:** 2020-04-28

**Authors:** Dahao Wu, Changzhen Wang

**Affiliations:** Department of Neurosurgery, Shandong Provincial ENT Hospital, Shandong Provincial ENT Hospital Affiliated to Shandong University, Jinan, China

**Keywords:** miR-155, glioma, PI3K/AKT signal pathway, proliferation, migration

## Abstract

**Objective:** Micro-RNA plays a critical role in the pathological process of gliomas. Previous research showed that the level of miR-155 was significantly increased in many cancers, including gliomas. However, the mechanism of glioma is still unknown.

**Method:** To investigate the regulatory function of miR-155 on glioma U87-MG cells and its effects on related signaling pathways. After transfection of miR-155 mimic and inhibitor, the level of miR-155 were applied to detect cell proliferation, apoptosis, senescence index, invasive ability and cell migration at different time points (0, 24, 24 h, respectively) by CCK8 assay, flow cytometry, β-galactosidase (β-gal) staining, transwell and scratch test, respectively. The effect of miR-155 on PI3K/AKT signal pathway was observed at meantime.

**Results:** Compared with the control group, after miR-155 mimic transfection, U87-MG cell viability, cell migration rate and invasiveness were increased, while apoptosis and senescence were significantly decreased, which was the opposite on miR-155 inhibitor transfection. The phosphorylation levels of miR-155, PI3K, AKT, PI3K, and AKT in U87-MG cells intervened with miR-155 mimic also increased significantly, while the levels of PTEN, Caspase-3, Caspase-9 mRNA, and protein declined significantly, with statistically significant difference. Meanwhile, compared with the control group, miR-155 inhibitor group were on the contrary.

**Conclusion:** The study indicated that miR-155 take charge a key function in regulating the proliferation, migration, and invasion of glioma U87-MG cells through PI3K/AKT signaling pathway, and has anti-glioma effects by inhibition of miR-155, which provided ideas for further clinical treatment of glioma patients.

## Introduction

Glioma is the primary malignant brain tumor of the central nervous system, which often occurs in children, adolescent, and middle-aged population (1). Although progress has been achieved in combined therapy, including chemotherapy, radiotherapy and surgical resection, the early diagnosis and prognosis of glioma patients have not been significantly improved (2, 3). The reason is that the infiltrative growth of glioma tissue leads to metastasis soon after the onset of symptoms, and there is no obvious effect target. Therefore, it is of great practical significance to find its directly and closely related target enabling early diagnosis of glioma, to explore its underlying mechanism of glioma, and to offer help for the development of new therapeutic targets.

MicroRNA (miRNA) is an endogenous non-coding small RNA with 20–22 nucleotides that involved in biological growth and development, and can binding the process of mRNA cleavage and translation inhibition (4, 5). There is growing research that miRNA regulate many cellular processes, including differentiation, metastasis, and proliferation (6). Latest research have shown that the abnormal levels of miRNA will change in the physiological process of tumors (7). Bai et al. indicated that miR-32 was down-regulated in non-small cell lung cancer which was closely related to patient progression and overall survival (8). Yang et al. indicated that miR-506 was down-regulated in clear cell renal cell carcinoma, and inhibited the growth of tumor cells by locating FLOT1 (9). Li et al. revealed that the function of miR-205 is to mediate tumor suppressor element binding protein 1 (CREB1) to inhibit the occurrence and development of colorectal cancer through targeting cAMP response (10). Glioma, like many tumor cells, is associated with numerous signaling pathways, including phosphatidylinositol 3 kinase/protein kinase B (PI3K/AKT) (11, 12). Domestic and overseas studies have confirmed that miR-21 can affect the growth and invasion and other biological behaviors of leukemic K562 cells, lung cancer cells and gastric cancer cells through PTEN/PI3K/AKT signaling pathway (13–15). Recently, it has been demonstrated that miR-34a mediates cisplatin-induced cell death via regulating PI3K/AKT/survivin pathway in gastric cancer (16), while miR-20a can induce radiation resistance effect by activating hepatoma PTEN/PI3K/Akt signal path (17). These multi-miRNA can exert biological functions in tumor diseases through PI3K/AKT. A new study reports, miR-155 is up-regulated in primary and secondary glioblastoma and promotes tumor growth by inhibiting GABA receptors, and blocking MIR155HG/miR-155 axis could inhibit mesenchymal transition in glioma (18). Gu et al. (19) found that melatonin inhibits the proliferation of glioma cells by inhibiting the expression of miR-155. The study of Qiu et al. showed that the deficiency of miR-155 in CD8+ T cells can achieve anti-glioma activity by inhibiting the proliferation and invasive activity of T cells (20). The study of Zhou et al. found that miR-155 may be a biomarker of glioma prognosis in the future by meta-analysis (21). Thus, it is speculated that miR-155 may also affect the development of glioma though the PI3K/AKT signaling pathway. Hence, the research will study this theory, so as to provide new ideas to treat the glioma.

## Materials and Methods

### Cell Strain

U87-MG was purchased from Chinese Academy of Sciences Cell Bank, cultured in 10% DMEM, FBS, 25 μg/ml streptomycin and 25 μg/ml penicillin. All cells were incubated in 5% CO_2_ cell incubator at 37°C.

### Transfection

miR-155 mimic or miR-155 inhibitor was purchased from RiboBio (Guangzhou, China). Liquid was changed and passaged every 48 h, and the cells in the logarithmic growth phase were taken for the experiment. In the experiment, the cells were cultured in OPTI-MEN serum-reducing medium and transfected with 50 nM Lipofectamine 2000 (Invitrogen) miR-155 mimic and miR-155 inhibitor, miR-NC (negative control). After 4 h, the cells were changed into complete medium and continued to be cultured. The transfection efficiency was examined by qPCR analysis. The sequence of miR-155 mimic is 5′- UCACAACCUCCUAGAAAGAGUAGA-3′. The sequence of miR-155 inhibitor is 5′-UCUACUCUUUCUAGGAGGUUGUGA-3′.

### Determination of Cell Viability

The cells was inoculated in 96-well culture plate with a density of 5 × 10^3^ cells per well. The cells were then transfected into the culture medium by miR-155 mimic or inhibitor. When infected and cultivated overnight, the cell growth status at different times (0, 24, 48, and 72 h) after transfection was measured by using CCK-8. Ten microliter CCK-8 solution was added to the 96-well plate and incubated for 1 h, followed by the detection of the value using enzyme labeling instrument (Bio-Rad laboratory) at 450 nm. The experiment was repeated 4 times. Cell viability (%) = (OD interference or overexpression group/OD control group 0 h) × 100 (%) (22).

### Western Blotting

Following the manufacturer's steps to extract the total protein (Sigma) from the cell line, all steps are carried out on ice. BCA protein assay kit determinate the concentration of total protein. The electrophoresis protein was heated to 100°C, incubated for 5 min, then using electrophoretic with SDS- polyacrylamide gel (120 v, 100 min). Afterwards, the protein separated from SDS gel was transferred to PVDF membrane (300 mA, 80 min). After the membrane transfer was completed, the target band was sealed with 5% TBS, and membrane was incubated with an anti-rabbit monoclonal anti-human GAPDH antibody (abcam catalog number ab8245), diluted overnight at 4°C with a dilution of 1:1,500. After incubation on the second day, the membrane was incubated with horseradish peroxidase to incubate the (HRP)-coupled polyclonal Goat anti-Rabbit IgG-HRP (Bioworld) second antibody (1:10,000). It was placed at room temperature for 1 h. The film has an enhanced chemiluminescence system and is imaged by X-ray film. The image is then quantized by Image J (NIH). Information about first antibody GAPDH (ab8245; abcam); PTEN (ab170941; abcam); AKT (ab8805; abcam); p-AKT (4060T; Cell Signaling Technology); PI3K (ab151549; abcam); p-PI3K (17366S; Cell Signaling Technology); Caspase-3 (ab13847; abcam); Caspase-9 (ab32539; abcam). The image is then quantized with Image J (NIH).

### Real Time Quantitative PCR (RT-PCR)

Total RNA was isolated using Trizol reagent (Invitrogen). A total of 500 ng RNA was reverse transcribed into cDNA using cDNA transcription kit (ABI). The enzyme was then transcribed at 16°C for 30 min, then incubated at 42°C for 30 min and inactivated at 85°C for 5 min. Rapid quantitative PCR was performed using SYBRH Select Master Mix (Invitrogen). Instead, the following parameters are applied for transcriptional response: 16°C, 30 min, 42°C, 30 min, and 85°C, 5 min. The QRT-PCR response was performed as follow: 95°C for 2 min, then 95°C for 15 s and 60°C for 40 cycles. Quantitative analysis was conducted by 2^−ΔΔCt^ method. The sequence of primers used is as follows: miR-155, 5′-ACACTCCAGCTGGTTAATGCTAATCGTGAT-3′ and 5′-TTAATGCTAATCGTGATAGGGGT-3′; U6, 5′-CTCGCTTCGGCAGCACA-3′ and 5′-AACGCTTCACGAATTTGCGT-3′; 18srRNA, 5′- CCTGGATACCGCAGCTAGGA-3′ and 5′-GCGGCGCAATACGAATGCCCC-3′.

### Detection of Apoptosis by Flow Cytometry

0.5 ml cell suspension was transferred from the cell culture plate (5 × 10 ^5^ cells) to a clean centrifuge tube. After adding 1.25 μl Annexin V-FITC and propidium iodide (PI) [keygen], the reaction was kept away from light for 15 min. The supernatant was removed by centrifugation at room temperature of 1,000 × g for 5 min. Add 10 μl Propidium Iodide the sample on the ice, kept away from the light. The stained cells were analyzed by flow cytometry (FAC Scan). The results were explained by Cell Quest software.

### β-Galactosidase Staining

The cells were washed with PBS or HBSS for once, then was fixed for 15 min by adding 1 ml β-galactosidase staining fixation solution. After removing PBS or HBSS, 1 ml staining fixative (Wuhan, beyotime) was incubated overnight at 37°C, observed under an ordinary optical microscope. Two hundred cells from 20 randomly selected fields of view were counted in each of three independently performed senescence assays.

### Cell Migration Experiment

First use the marker pen to draw a horizontal line evenly on the back of the 6-well plate across the hole, at intervals of about 0.5–1 cm. Fifty microliter of fibronectin (FN) of 10 μg/ml was placed overnight in a refrigerator at 4°C, followed by steril ization by adding RPMI-1640 medium with 10% FBS under ultraviolet light for 2 h. Cells in logarithmic phase with about 1 × 10^6^/ml concentration were made into suspension and inoculated evenly in 6-well culture plate, routinely cultured in 5% CO_2_ incubator at 37°C. The cells were changed the liquid after growing into a monolayer, and then treated with mitomycin for 1 h to inhibit cell division. With scratch on the cell plate with the head of a 10 μl fluid transfer gun, PBS solution was washed 2–3 times to remove the scraped cells. Samples were taken at 0 and 48 h after scratching, with photos taken. The healing condition of scratches was observed under inverted microscope. Image J was used to measure the scratch width of cells at any eight sites in each group at the same point-in-time, and the cell migration distance ratio = (0 h-other time points)/0 h was calculated to reflect the movement and migration ability of U86 cells, and the data were counted.

### Transwell Experiment

Matrigelwas diluted with precooled serum-free medium at 1:3 and 40 μl of it was added to the precooled Transwell chamber. After Matrigel was incubated at 37°C for 2 h, The excess fluid in the chamber was sucked away, adding serum-free medium 100 and 600 μl into the upper and lower chambers, respectively, balanced overnight at 37°C. On the second day after transfection of microR-155 mimic and miR-155 inhibitor, 1 × 10^5^ cells were counted and resuspended with 100 μl serum-free DMEM-F12 medium, added to the upper chamber of Transwell, while the complete medium of 600 μl was added in the lower chamber. After incubating at 37°C, 5% CO_2_ for 24 and 48 h, the cells in the upper chamber were removed with cotton swabs after being taken out form the chamber, fixed with 4% paraformaldehyde for 15 min, washed once for 15 min with PBS, and stained with crystal violet for 10 min, washed with PBS once again. Detection of whether the cells passed through the holes were tested. If so, took photos and statistics.

### Statistical Analysis

The experimental measurements were repeated at least three times. All data are expressed as mean ± standard deviation (SD). All data were analyzed with SPSS 18.0, *t*-test or one-way analysis of variance. *P* < 0.05 is considered to indicate a statistically significant difference.

## Result

### Transfection Efficiency of miR-155 in U87-MG Cells

**Figure 1:**
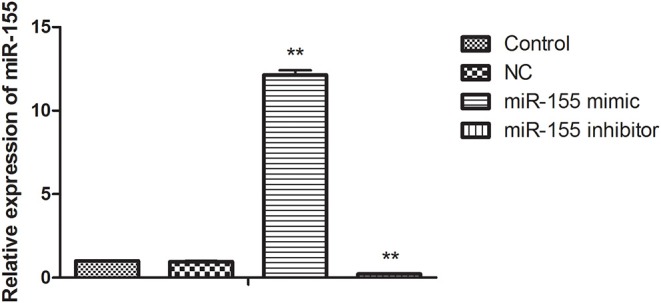
Change of miR-155 expression level after transfection. **P < 0.01 vs. control group.

In order to find the biological function of miR-155 in glioma, U87-MG cells were transfected with NC, miR-155 mimic and miR-155 inhibitor, through function acquisition and deletion experiment. The expression of miR-155 in miR-155 mimic group was significantly higher than which in NC group, while the level of miR-155 in miR-155 inhibitor group was significantly declined (*P* < 0.05) (Figure 1).

### Effect of miR-155 on Cell Viability

**Figure 2:**
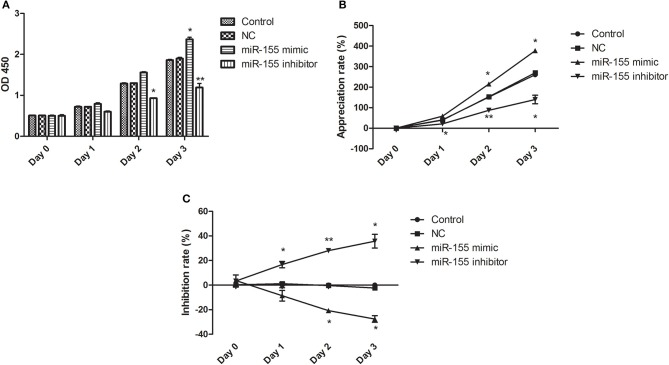
Effect of miR-155 on the activity of U87-MG cells. **(A)** Cell sensitivity of U87MG treated with different methods. **(B)** Cell proliferation of U87MG treated with different methods. **(C)** Cell inhibition of U87MG treated with different methods. *P < 0.05 vs. control group, **P < 0.01 vs. control group.

After transfection of NC, miR-155 mimic and miR-155 inhibitor, CCK8 assay was applied to detect the activity of glioma U87-MG cells. NC had no significant effect on the cell viability at different time points, the miR-155 mimic group could significantly increase the cell viability of U87-MG at 48 and 72 h, but there was no significant change at 24 h, while the miR-155 inhibitor group could inhibit the cell viability of U87-MG at 24, 48 and 72 h (*P* < 0.05) (Figures 2A–C).

### Effect of miR-155 on Apoptosis of U87-MG Cells

**Figure 3:**
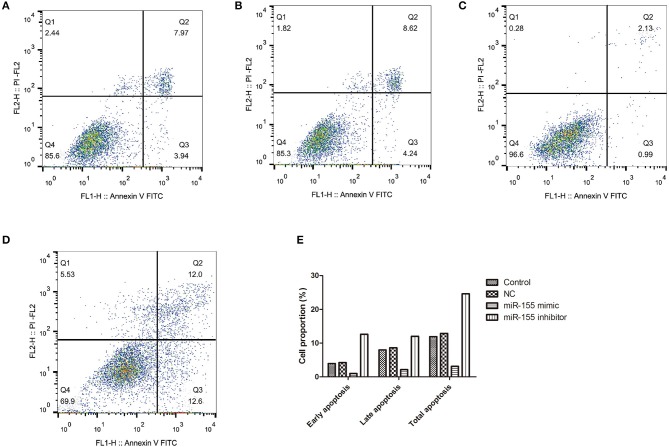
Effect of miR-155 on apoptosis of U87-MG cells. **(A)** Apoptosis in Control group. **(B)** Apoptosis in NC group. **(C)** Apoptosis in miR-155 mimic group. **(D)** Apoptosis in miR-155 inhibitor group. **(E)** Early, late and total apoptosis.

To explore the biological function of miR-155 on U87-MG cells, different apoptotic stages were detected by flow cytometry with two kinds of dyes. The results showed that NC had no significant effect on apoptosis, while miR-155 mimic group could significantly inhibit the early and late apoptosis of U87-MG cells, with the total number of apoptotic cells decreased, whereas miR-155 inhibitor group could significantly promote the early and late apoptosis of U87-MG cells (*P* < 0.05) (Figures 3A–E).

### Effect of miR-155 on Senescence of U87-MG Cells

**Figure 4:**
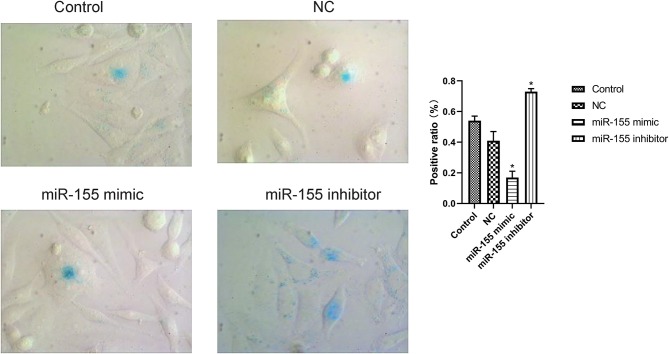
Effect of miR-155 on senescence of U87-MG cells detected. *P < 0.05.

The results of β-gal staining suggested that the number of senescent cells and senescence index in miR-155 mimic group were decreased, while those in miR-155 inhibitor group were significantly increased (*P* < 0.05). Two hundred cells from 20 randomly selected fields of view were counted in each of three independently performed senescence assays (Figure 4).

### Effects of miR-155 on Invasion and Migration of U87-MG Cells

**Figure 5:**
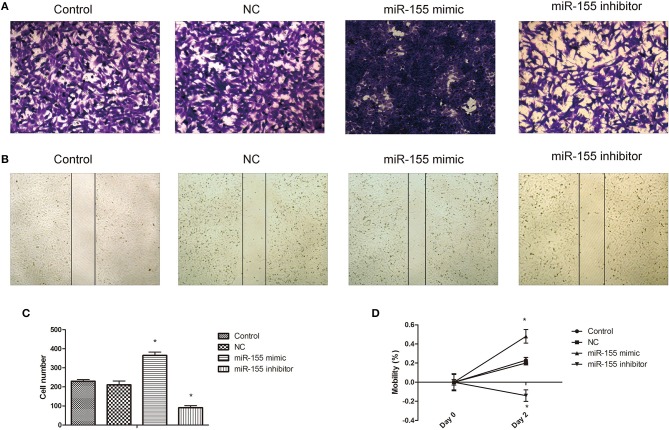
Effect of miR-155 on invasion and migration of U87-MG cells. **(A)** Effect of miR-155 on migration of U87-MG cells. **(B)** Effect of miR-155 on invasion of U87-MG cells. **(C)** Histogram statistics of cell invasion rate. **(D)** Histogram statistics of cell mobility; *P < 0.05 vs. control group.

Transwell and cell scratch assay were used to detect the effect of miR-155 transfection on the invasion and migration. The results show that the invasion and migration ability of NC group was not significantly affected, but the invasion and migration ability of miR-155 mimic group was increased, while that of miR-155 inhibitor group was significantly decreased (*P* < 0.05) (Figures 5A–D).

### Effect of miR-155 on the Expression of PTEN, Caspase-3, and Caspase-9 in U87-MG Cells

**Figure 6:**
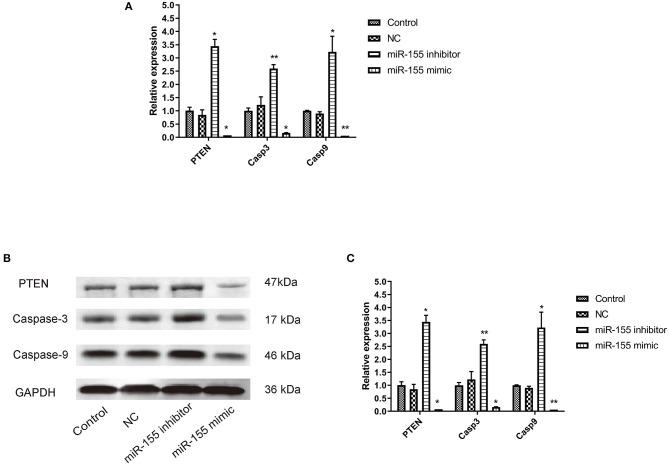
Effect of miR-155 on the expression of PTEN, Caspase-3 and Caspase-9 in U87-MG cells. **(A)** Effect of miR-155 on the expression of PTEN, caspase-3 and caspase-9 mRNA in U87-MG cells. **(B)** Effect of miR-155 on PTEN, caspase-3 and caspase-9 protein expression in U87-MG cells. **(C)** Gray statistical value of WB experiment; *P < 0.05 vs. control group, **P < 0.01 vs. control group.

The corresponding apoptotic factors, PTEN, Caspase-3, and Caspase-9, were detected after transfection. RT-PCR and Western blot showed that the mRNA and protein levels of gene in NC group had no significant change, while the mRNA and protein levels of PTEN, Caspase-3, and Caspase-9 in miR-155 mimic group were significantly down-regulated and the those in miR-155 inhibitor group were higher (*P* < 0.05) (Figures 6A–C).

### Effect of miR-155 on the Expression of Proteins Related to PI3K/AKT Signaling Pathway in U87-MG Cells

**Figure 7:**
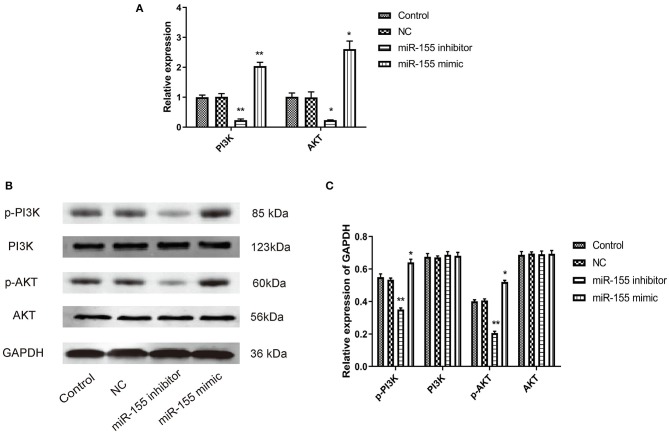
Effect of miR-155 on the expression of proteins related to PI3K/AKT signaling pathway in U87-MG cells. **(A)** Effect of miR-155 on the expression of PI3K and AKT mRNA in U87-MG cells. **(B)** Effect of miR-155 on PI3K and AKT protein expression in U87-MG cells. **(C)** Gray statistical value of WB experiment; *P < 0.05 vs. control group, **P < 0.01 vs. control group.

PI3K/AKT signal pathway is abnormally activated in the pathological process of tumor, but the effect of miR-155 on this signal pathway is still unclear. Whether miR-155 can function in glioma by regulating PI3K/AKT signal pathway remains to be further studied. After transfection of NC, miR-155 mimic and miR-155 inhibitor into U87-MG cells, RT-PCR and Western blot detected the related genes of the signaling pathway. It was found that the expression of mRNA and protein in NC group had no significant change, and the total protein of PI3K and AKT in miR-155 mimic group had no significant change, but the levels of mRNA and phosphorylation were significantly increased. In miR-155 inhibitor group, the total protein of PI3K and AKT had no significant change, while the levels of mRNA and phosphorylation were significantly lower than those of control group (*P* < 0.05) (Figures 7A–C).

## Discussion

Glioma remains a major public health challenge and poses a high risk of morbidity and mortality associated with brain tumors (23) and a serious threat to human life and property. Currently, the molecular mechanism of promoting glioma is still unclear. Exposure to high doses of ionizing radiation and inheritance of high-risk gene mutations are the only identified risk factors for gliomas (24, 25). Therefore, it is of great value to further reveal the pathogenesis of glioma cancer and explore new therapeutic drugs and methods. Previous research had shown that epigenetic modification may be a new method for the treatment of gliomas (26).

MicroRNA plays an critical biological role in many aspects of tumor (27). miR-155 is usually expressed in many cancers and participates in life activity, including migration and invasion (28). Ji et al. confirmed that the level of overexpression of miR-155 in renal clear cell carcinoma can work as carcinogenic function by targeting FOXO3a (29). Zhu et al. found that down-regulation of miR-155 in diffuse large B-cell lymphoma may promote cell cycle arrest and apoptosis (30).

Latest reports have shown that miR-155 also serves certain biological function in the progression of gliomas. Sun et al. suggested that the level of miR-155 was up-regulated in glioma patients, accompanied by high pathological grade (31). Cao et al. confirmed that MALAT1 can increase cell viability by inhibiting miR-155 and promoting FBXW7 expression, thus inhibiting the occurrence and development of glioma (32). Wu et al. found that blocking the MIR155HG/miR-155 molecular axis can inhibit the interstitial transformation of glioma (33). However, the mechanism of miR-155 in glioma is still unknown. In the current study, biological function of miR-155 in glioma cell line U87-MG was first determined. The effects of miR-155 on cell proliferation apoptosis infiltration and migration were detected by CCK8 flow cytometry transwell test and cell scratch test. The study demonstrated that miR-155 mimic significantly promoted the proliferation, infiltration, and migration and reduced the apoptosis and senescence of glioma cells. We also found that miR-155 promoted the growth of glioma cells *in vivo*.

Apoptosis-related genes strictly regulate cell apoptosis (34), in which Caspase is a cascade of cysteine-aspartic acid-specific proteases (35). The cascade pathway of cell apoptosis mediated by its protein family is activated by the action of multiple factors, and finally leads to cell apoptosis through a series of cascade reactions (36). Caspase-9 is the initiator of the Caspase cascade reaction. After the death receptor receives the signal, it causes the activation of pro-Caspase-9 and initiates the Caspase cascade reaction, activating its downstream Caspase-3. Caspase-3 is the terminal cleavage enzyme of apoptosis, which is related to DNA repair and gene integrity monitoring (37). To study the mechanism of miR-155 in apoptosis, the research further explored the effect of miR-155 on the mRNA and protein expression of Caspase-3 and Caspase-9 in U87-MG cells. The study indicated that overexpression of miR-155 significantly down-regulate the expression of Caspase-3 and Caspase-9, thus promotes the proliferation and inhibit apoptosis.

The physiological processes of tumor cell growth, proliferation, differentiation, apoptosis, migration, invasion, and angiogenesis are cross-regulated by a variety of signal pathways, including PI3K/AKT signal pathway (38). This signaling pathway, also known as anti-apoptotic pathway, transmits upstream stimuli to phosphatidylinositol diphosphate (PIP2), phosphorylating PIP2 to form inositol triphosphate (PIP3), transmits the signal to AKT, to promote the phosphorylation of the serine/threonine site of AKT, and transmits the signal to the nucleus, to then regulate the expression and translation of downstream target genes (39). However, the upstream signal of PI3K, including PTEN, can promote the dephosphorylation of PIP2 by PIP3, and inhibit the activity of AKT, which plays a negative regulatory role in the PI3K/AKT signaling pathway (40). Huang et al. found that miR-155 activated AKT activity by directly targeting the positive and negative regulator p85α of PI3K-Akt pathway (41). Xue et al. found that over expression of miR-155 changes AKT signaling by inhibiting SHIP1 expression (42). Fu et al. Showed that miR-155-5p further activated the PI3K/Akt pathway by inhibiting PTEN and inhibiting apoptosis (43). In addition, PTEN is also one of the downstream target genes of miR-155, and its expression is low or even deficient in various tumors including gliomas (44). The occurrence and development of hepatocellular carcinoma induced by miR-155 is closely associated with the expression of Caspase3, Caspase-9, and PTEN (43). After transfection, the effect of miR-155 on the activation of PI3K/AKT signal pathway was observed. The results suggested that the total protein level of PI3K and AKT in miR-155 inhibitor group did not change significantly, but its mRNA and phosphorylation levels decreased significantly, whereas the result of miR-155 mimic was just the opposite. The data suggested that miR-155 could activate PI3K/AKT signal pathway to regulate the expression of PTEN, Caspase-3, and Caspase-9, and finally promote cell proliferation and induce cell apoptosis.

In short, our research found that the high level of miR-155 in gliomas can promote the proliferation, invasion and migration of U87-MG glioma cells, inhibit their senescence and apoptosis, and activate the PI3K/AKT signal pathway, thus aggravating the occurrence and development of gliomas. The findings of these results have important practical significance for miR-155 to be a and potential therapeutic target for the diagnosis and treatment of gliomas.

## Data Availability Statement

All datasets generated for this study are included in the article/supplementary material.

## Author Contributions

DW and CW: draft manuscript, write article, modify manuscript, statistical data, analyze data, experiment operation, and agree to publish manuscript.

## Conflict of Interest

The authors declare that the research was conducted in the absence of any commercial or financial relationships that could be construed as a potential conflict of interest.
